# Admission factors associated with intensive care unit readmission in critically ill oncohematological patients: a retrospective cohort study

**DOI:** 10.5935/0103-507X.20160011

**Published:** 2016

**Authors:** Cinthia Mendes Rodrigues, Ellen Maria Campos Pires, Jorge Patrick Oliveira Feliciano, Jose Mauro Vieira Jr., Leandro Utino Taniguchi

**Affiliations:** 1 Research and Education Institute, Hospital Sirio-Libanes - São Paulo (SP), Brazil.; 2 Emergency Medicine Discipline, Hospital das Clínicas, Faculdade de Medicina, Universidade de São Paulo - São Paulo (SP), Brazil.

**Keywords:** Patient readmission, Oncology service, hospital, Risk factors, Intensive care units

## Abstract

**Objective:**

The purpose of our study was to determine the admission factors associated with intensive care unit readmission among oncohematological patients.

**Methods:**

Retrospective cohort study using an intensive care unit database from a tertiary oncological center. The participants included 1,872 critically ill oncohematological patients who were admitted to the intensive care unit from January 2012 to December 2014 and who were subsequently discharged alive. We used univariate and multivariate analysis to identify the admission risk factors associated with later intensive care unit readmission.

**Results:**

One hundred seventy-two patients (9.2% of 1,872 oncohematological patients discharged alive from the intensive care unit) were readmitted after intensive care unit discharge. The readmitted patients were sicker compared with the non-readmitted group and had higher hospital mortality (32.6% versus 3.7%, respectively; p < 0.001). In the multivariate analysis, the independent risk factors for intensive care unit readmission were male sex (OR: 1.5, 95% CI: 1.07 - 2.12; p = 0.019), emergency surgery as the admission reason (OR: 2.91, 95%CI: 1.53 - 5.54; p = 0.001), longer hospital length of stay before intensive care unit transfer (OR: 1.02, 95%CI: 1.007 - 1.035; p = 0.003), and mechanical ventilation (OR: 2.31, 95%CI: 1.57 - 3.40; p < 0.001).

**Conclusions:**

In this cohort of oncohematological patients, we identified some risk factors associated with intensive care unit readmission, most of which are not amenable to interventions. The identification of risk factors at intensive care unit discharge might be a promising approach.

## Abstract (pt)

**Objetivo:**

Determinar os fatores na admissão associados a readmissões na unidade de terapia intensiva em pacientes onco-hematológicos.

**Métodos:**

Estudo retrospectivo de coorte utilizando a base de dados de uma unidade de terapia intensiva de um hospital oncológico terciário. Os participantes foram 1.872 pacientes onco-hematológicos graves admitidos à unidade de terapia intensiva entre janeiro de 2012 e dezembro de 2014, e que sobreviveram e receberam alta da unidade. Utilizamos análises univariada e multivariada para identificar os fatores de risco na admissão associados com readmissões mais tarde à unidade de terapia intensiva.

**Resultados:**

Dos 1.872 que sobreviveram e receberam alta da unidade de terapia intensiva, 172 (9,2%) pacientes foram readmitidos após terem recebido alta da unidade. Os pacientes readmitidos tinham enfermidade mais grave, quando comparados aos do grupo que não foi readmitido, além de taxa de mortalidade hospitalar mais elevada (32,6% *versus* 3,7%, respectivamente; p < 0,001). Na análise multivariada, os fatores de risco independentes para readmissão à unidade de terapia intensiva foram: sexo masculino (OR: 1,5; IC95%: 1,07 - 2,12; p = 0,019), cirurgia de emergência como causa da admissão (OR: 2,91; IC95%: 1,53 - 5,54; p = 0,001), maior tempo de permanência no hospital antes da transferência para a unidade de terapia intensiva (OR: 1,02; IC95%: 1,007 - 1,035; p = 0,003) e ventilação mecânica (OR: 2,31; IC95%: 1,57 - 3,40; p < 0,001).

**Conclusão:**

Nesta coorte de pacientes onco-hematológicos foram identificados alguns fatores de risco associados à readmissão na unidade de terapia intensiva, a maioria não passível de intervenção. A identificação dos fatores de risco na alta da unidade de terapia intensiva pode ser uma abordagem promissora.

## INTRODUCTION

After recovery from critical illness, some patients are susceptible to new complications, many of which require intensive care unit (ICU) readmission. This is associated with increased mortality and longer hospital stays.^(1,2)^ The early identification of patients at risk for ICU readmission might facilitate appropriate resource allocation to prevent increases in both morbidity and mortality. Individualized healthcare planning that includes decisions about the right moment for discharge and the proper discharge facility (e.g., the ward or intermediate care unit) could be devised for high-risk patients. Previous data have suggested that some deaths after ICU discharge are avoidable.^(3)^

Some risk factors associated with ICU readmission have been identified, including older age, severity of illness, comorbidities, after-hours discharge, emergency surgery, and transfer to a high-dependency unit.^(1,2,4,5)^ However, these previous studies evaluated a general population of critically ill patients and not specifically oncohematological patients. This patient population has increased in ICU over the years. New treatments with better results have increased the chances of cure. Nevertheless, associated treatment toxicities and immunosuppression have also increased ICU admissions.^(6)^ Because comorbidities related to cancer and its treatment are long-lasting after ICU discharge, cancer patients are particularly prone to readmission and the associated morbimortality.

The objective of this study was to identify at the first intensive care unit admission some risk factors associated with later intensive care unit readmission among critically ill oncohematological patients.

## METHODS

This is a retrospective cohort analysis of patients admitted to the 30-bed, mixed medical-surgical ICU of *Hospital Sírio-Libanês*, a private tertiary hospital with a dedicated oncology unit in São Paulo, Brazil. Cardiac surgical patients are managed in a separate unit within our hospital. Because our ICU has an "open format" model, admission and discharge decisions are made after discussions between the patient's attending physician and the intensive care physician. There is no formal follow-up by the ICU team after discharge. The hospital has an intermediate care unit with 40 beds, the 24-h presence of an intensivist, and a higher nurse-to-patient ratio than the ward. The study was approved by the local institutional ethics committee, which waived informed consent because of the observational design of the study (CAAE: 42763115.7.0000.5461).

Our analysis used de-identified administrative data that were prospectively collected at ICU admission in a software database (Sistema Epimed^TM^; www.epimedmonitor.com) by one of the authors. The study population consisted of all consecutive adult patients over 18 years of age who were admitted between January 1, 2012, and December 31, 2014 with an oncohematological condition. The definition of oncohematological condition was active cancer (current curative or palliative chemotherapy, radiotherapy, immunotherapy, or surgery) or bone marrow transplantation in the previous 12 months. Cancer that entered remission without therapy within the previous 6 months was not considered active. The exclusion criteria were ICU length of stay (LOS) less than 12 h (to exclude patients admitted for minor procedures, such as cardiac catheterization), pregnancy, and patient unsuitability for ICU readmission (death on the unit or transfer to another hospital or to palliative care).

The data recorded included age, sex, Simplified Acute Physiology Score 3 (SAPS 3),^(7,8)^ referring facility, admission diagnosis, surgical procedures before admission, the presence and type of comorbidities, the length of hospital stay before ICU admission, resource use during ICU stay (mechanical ventilation, vasoactive drugs, or renal replacement therapy) and hospital mortality. Sepsis was defined according to a previous consensus definition.^(9)^ Readmission was defined as the ICU admission of a patient who had been previously admitted to the ICU during the same hospitalization. If multiple readmission episodes occurred, only the first was considered for the present analysis.^(2,10)^

### Statistical analysis

The data were analyzed using IBM Statistical Package for Social Science (SPSS), for Windows, Version 20.0 (IBM Corp., Armonk, NY, USA). Normality of distribution was verified with the Kolmogorov-Smirnov test for continuous variables. Data are presented as the mean (SD) or median [25^th^ percentile - 75^th^ percentile] for parametric and nonparametric variables, respectively. Categorical variables are presented as rates or percentages. Comparisons of parametric variables between groups were performed with an unpaired Student's *t*-test, and comparisons within groups were performed with a paired Student's *t*-test; non-parametric variables were compared within groups using a Wilcoxon signed-rank test and between groups using a Mann-Whitney test. All statistics were two-tailed, and a p-value < 0.05 was considered statistically significant.

We performed a multivariate logistic regression analysis with ICU readmission as the dependent factor. Variables with a p-value < 0.1 in the univariate analysis were included in the logistic model. Multicollinearity was excluded using the variance inflation factor before modeling.^(11,12)^ The model was refined using the backward stepwise likelihood ratio method, and the least significant variable at each step was excluded if its associated significance level was greater than 0.05. All of the included variables had less than 2% missing data, and no imputation was performed for missing values. The calibration and discrimination of the prediction model were evaluated with the Hosmer-Lemeshow goodness-of-fit test and the area under the curve (AUC), respectively.

## RESULTS

**Figure 1:**
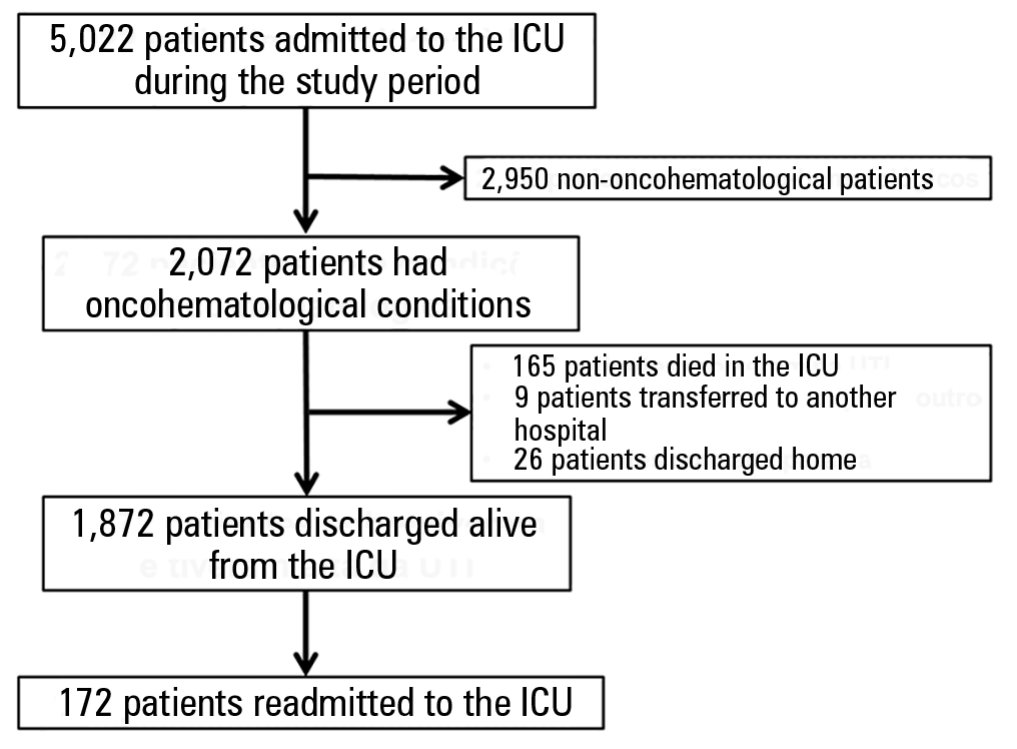
Patient flow diagram of the study. ICU - intensive care unit.

**Figure 2:**
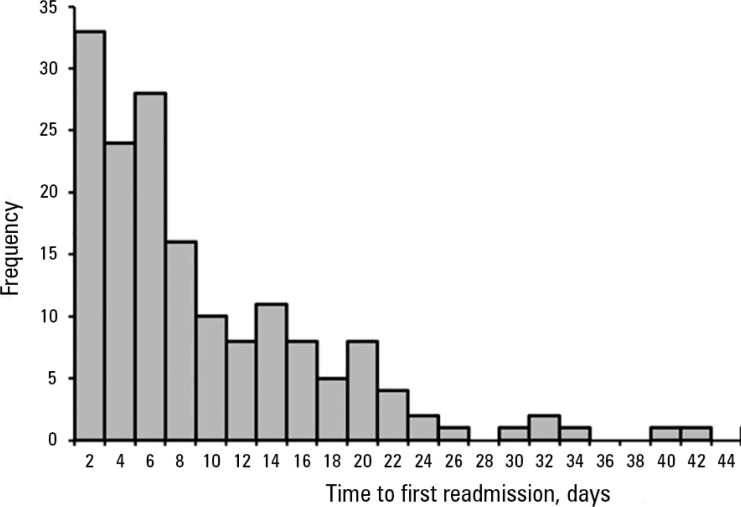
Histogram of time to first readmission after intensive care unit discharge.

Of the 5,022 patients admitted to the ICU during the study period, 2,072 patients had oncohematological conditions (41.3%), and 165 (8.0%) of these patients died in the ICU during the first admission. Of the remaining 1,907 patients, nine were transferred to another hospital, and 26 were discharged home. Finally, 1,872 patients were discharged alive from the ICU and composed the study group (Figure 1). Readmission occurred for 9.2% of discharged patients after a median of 6.5 days [4-14 days] after discharge (Figure 2). The study group characteristics are presented in table 1.

**Table 1:** Patient characteristics at first intensive care unit admission

	**All patients**	**No readmission**	**Readmission**	p value*
	**(N = 1,872)**	**(N = 1,700)**	**(N = 172)**	
Age (SD) (years)	62.3 (16.5)	62.2 (16.6)	63.1 (15.4)	0.58
Male	1,046 (55.9)	935 (55.0)	111 (64.5)	0.013
SAPS 3	37 [29 - 48]	36 [28 - 48]	44 [35 - 52]	< 0.001
Admission type				< 0.001
Medical	582 (31.1)	508 (29.9)	75 (43.6)	
Emergency surgery	71 (3.8)	58 (3.4)	14 (8.1)	
Elective surgery	1,217 (65.0)	1134 (66.7)	83 (48.3)	
Admission source				< 0.001
Ward	195 (10.4)	150 (8.8)	44 (25.6)	
Emergency room	253 (13.5)	235 (13.8)	18 (10.5)	
Operating room	1,280 (68.4)	1,187 (69.8)	95 (55.2)	
Intermediate care	45 (2.4)	43 (2.5)	10 (5.8)	
ICU discharge during weekends	432 (23.1)	384 (22.6)	48 (27.9)	0.12
Length of hospital stay before ICU admission (median days)	1 [0 - 2]	1 [0 - 2]	1 [1 - 5]	< 0.001
Neoplasia subtype**				0.016
Locoregional	1,336 (71.4)	1232 (72.5)	104 (60.5)	
Metastatic	401 (21.4)	357 (20.9)	45 (26.2)	
Hematological	170 (9.1)	141 (8.3)	29 (16.9)	
Non-oncohematological comorbidities				0.08
0	1,666 (89.0)	1,521 (89.5)	144 (83.7)	
1	187 (10.0)	162 (9.5)	27 (15.7)	
≥ 2	19 (1.0)	17 (1.0)	1 (0.6)	
Admission diagnosis				
Sepsis	97 (5.2)	83 (4.9)	14 (8.1)	0.07
Shock	232 (12.4)	209 (12.3)	23 (13.4)	0.69
Respiratory failure	82 (4.4)	70 (4.1)	13 (7.6)	0.034
Neurological disturbance	109 (5.8)	95 (5.6)	14 (8.1)	0.17
Mechanical ventilation at admission	262 (14.0)	218 (12.8)	45 (26.2)	< 0.001
Vasoactive drug at admission	494 (26.4)	442 (26.0)	53 (30.8)	0.19
Dialysis at admission	56 (3.0)	49 (2.9)	8 (4.7)	0.21

SD - standard deviation; SAPS - Simplified Acute Physiology Score 3; ICU - intensive care unit.

* p value for comparison between non-readmitted and readmitted groups.

** Seven patients had concomitant hematological and solid metastatic cancer, and 30 had concomitant hematological and solid locoregional cancer. The results are expressed as number (%) or median [25 - 75%].

At the first ICU admission, the patients who were later readmitted were sicker, had a non-elective surgical reason for admission, were more frequently male, were admitted from the ward, were admitted after longer hospital LOSs, had hematological cancer (but a lower frequency of solid locoregional cancer), were admitted for respiratory failure, and required mechanical ventilation more frequently at admission compared with the patients who were not readmitted. Of note, readmission was significantly associated with higher hospital mortality compared with non- readmission (32.6% versus 3.7%; p < 0.001). ICU discharges on weekends did not differ between the groups (22.6% versus 27.9%; p = 0.12).

Compared with the first admission, on readmission, the patients had higher SAPS 3, often had unplanned admissions (81.9%), were readmitted from the ward or intermediate care unit, and had a higher incidence of respiratory failure or neurological disturbance as the reason for readmission (Table 2).

**Table 2:** Comparison of readmitted patients at first intensive care unit admission and at readmission

	**First admission**	**Readmission**	p value*
	**(N = 172)**	**(N = 172)**	
SAPS 3	44 [35 - 52]	50 [42.3 - 59]	< 0.001
Admission type			< 0.001
Medical	75 (43.6)	121 (70.1)	
Emergency surgery	14 (8.1)	20 (11.8)	
Elective surgery	83 (48.3)	31 (18.1)	
Admission source			< 0.001
Ward	44 (25.6)	67 (38.9)	
Emergency room	18 (10.5)	---	
Operating room	95 (55.2)	49 (28.5)	
Intermediate care	10 (5.8)	36 (20.8)	
Admission diagnosis			
Sepsis	14 (8.1)	18 (10.4)	0.47
Shock	23 (13.4)	33 (19.4)	0.14
Respiratory failure	13 (7.6)	26 (15.3)	0.029
Neurological disturbance	14 (8.1)	28 (16.0)	0.029
Mechanical ventilation at admission	45 (26.2)	45 (26.4)	0.95
Vasoactive drug at admission	53 (30.8)	57 (33.3)	0.63
Dialysis at admission	8 (4.7)	5 (2.8)	0.39

SAPS - Simplified Acute Physiology Score 3.

* Paired comparison between readmission and first intensive care unit admission. The results are expressed as number (%) or median [25 - 75%].

In the multivariate analysis (Table 3), the independent risk factors for ICU readmission were male sex (odds ratio (OR) = 1.5, 95% confidence interval (CI): 1.07 - 2.12; p = 0.019), emergency surgery as the admission reason (OR = 2.91, 95%CI: 1.53 - 5.54; p = 0.001), longer hospital LOS before ICU transfer (OR = 1.02, 95%CI: 1.007 - 1.035; p = 0.003), and mechanical ventilation (OR = 2.31, 95%CI: 1.57 - 3.40; p < 0.001). The Hosmer-Lemeshow test was non-significant for the final model (p = 0.12). The AUC was 0.69 (95%CI: 0.66 - 0.74; p < 0.001).

**Table 3:** Factors associated with intensive care unit readmission in a multivariate analysis

**Parameter**	**OR**	**95%CI**	**p value**
Male sex	1.5	1.07 to 2.12	0.019
Emergency surgery	2.91	1.53 to 5.54	0.001
Length of hospital stay before ICU admission (days)	1.02	1.007 to 1.035	0.003
Mechanical ventilation at ICU admission	2.31	1.57 to 3.40	< 0.001

OR - odds ratio; CI - confidence interval; ICU - intensive care unit. Area under the receiver operating curve for predicted mortality (95%CI): 0.69 (0.66 to 0.74), p < 0.001. Hosmer- Lemeshow χ^2^ p = 0.12.

## DISCUSSION

In this cohort of critically ill oncohematological patients, we observed a readmission rate of 9.2%, mostly for unplanned episodes. Some differences were observed on initial admission between the patients who were readmitted to the ICU compared with those who were not readmitted. The most relevant finding was that readmission was associated with a tenfold increase in mortality. Male sex, emergency surgery, longer LOS before ICU transfer, and mechanical ventilation were independently associated with ICU readmission.

There is a paucity of research specifically addressing oncohematological patients and ICU readmission. Song et al. published a retrospective cohort analysis of patients discharged after thoracic oncological surgery and described a readmission rate of 8.6%, which is similar to our finding. However, those authors only enrolled surgical patients with lung or esophageal cancer, which limits the generalizability of their findings.^(13)^ Although other studies have demonstrated rates that are comparable to ours in general populations of critically ill medical patients,^(14)^ our rate is higher than those of most previously published studies.^(1,2,4,5,10)^ In fact, a recent systematic review of 58 studies suggested that readmission rates are generally between 4% and 6% for critically ill patients.^(15)^ The reasons for our comparably high readmission rate might be related to differences in inclusion criteria. For example, the systematic review by Hosein et al. excluded articles that described discharge from a high dependency or step-down unit,^(15)^ whereas we did not. However, another reason might be that oncohematological patients are more susceptible to post-ICU complications that require readmission. Treatment-related immunosuppression, cancer-associated malnutrition, invasive procedures, repeated surgeries, and increased thrombotic tendency are some factors that make oncological patients frailer and prone to readmission.

We observed a tenfold hospital mortality increase in patients who were readmitted to the ICU. Higher readmission rates are usually associated with increased rates of mortality and morbidity;^(1,2)^ therefore, strategies to decrease readmission rates are advisable. One option is to recognize high-risk subgroups by identifying risk factors that require differentiated attention. In our multivariate analysis, male sex, emergency surgery, longer LOSs before ICU admission, and mechanical ventilation were independently associated with readmission. Most of these risk factors translate to a higher severity of illness or a previous burden of chronic health problems. Similar risk factors have been reported previously^(1,2,14)^ and have recently been summarized in the Stability and Workload Index for Transfer (SWIFT) score.^(16)^ However, this score includes arterial blood gas analysis, which is not routinely performed in most patients close to the time of deciding their discharge from the ICU. Even if this test is performed, the presence of hypoxemia and/or hypercapnia denotes pulmonary dysfunction. Residual organ dysfunction at ICU discharge has been previously associated with readmission^(14,17)^ and long-term mortality.^(18)^ However, again, the presence of organ dysfunction at discharge indicates a state of enduring vulnerability that is usually related to greater severity of illness. This must be taken into account to plan actions such as determining the discharge facility (e.g., an intermediate care or high-dependency unit). Intermediate care units are generally viewed as more appropriate for some patients because patient management includes higher nurse-to-patient ratios and more intensive monitoring compared with the general ward.^(19)^ Nevertheless, the populations that will benefit most remain unknown because transfer to such facilities might not result in reduced mortality or hospital LOS.^(20)^

Because some deaths associated with readmission are thought to be preventable,^(3)^ readmission rates are usually seen as a quality metric that is even subject to financial penalties.^(21)^ However, recent studies have cast some doubts on this. Luthi et al. did not find any association between readmission and quality of care in patients with heart failure.^(22)^ Fischer et al. conducted a recent systematic literature review and observed that many methodological issues preclude an unbiased estimate of in-hospital quality of care using readmission rates.^(23)^ Incorrect case-mix adjustment is a major problem, as recently demonstrated by Kramer et al.^(10)^ After adjustment for in-hospital mortality, those authors did not observe significant differences in standardized mortality or lengths of stay between ICUs with high rates of readmission compared with units that had moderate or low rates. Thus, comparisons between ICUs, even those within the same hospital, should be interpreted with caution. However, the use of this metric as a quality indicator has been suggested in a recent European Society of Intensive Care Medicine report.^(24)^

Our study has some limitations. First, it is a single-center retrospective analysis of a private tertiary oncology center, which might limit the generalizability of our findings. Second, we only studied admission factors associated with later readmission. Patient condition upon ICU discharge is likely a better predictor of readmission, especially in tandem with certain laboratory data (e.g., C-reactive protein). However, our final model has an AUC similar to those of previous reports^(14)^ or external validations of SWIFT scores.^(16)^ Finally, we used data collected for administrative purposes, which are usually missing more clinically relevant information than data collected for observational studies. However, our data are readily available and might provide relevant information for future research (for example, by using a population database to estimate the burden of a particular condition, such as sepsis^(25)^. Because there is a lack of ICU readmission studies among oncohematological patients, our data might provide useful information for prospective studies of the issue (e.g., the role of biomarkers as risk factors for readmission^(26)^.

## CONCLUSION

In summary, in our cohort of oncohematological patients discharged alive from the intensive care unit, male sex, emergency surgery, longer length of stay before intensive care unit admission, and mechanical ventilation were identified as independent risk factors for readmission. Because these characteristics were identified at the first admission, they should also be evaluated at intensive care unit discharge.
